# National Epilepsy Awareness Month — November 2014

**Published:** 2014-11-07

November is National Epilepsy Awareness Month. Epilepsy is a brain disorder characterized by recurrent seizures and affects an estimated 2.3 million adults and 450,000 children in the United States (1,2). Eighty-seven percent of parents of children with epilepsy have reported needing care coordination, and of these, 45% had unmet needs (3).

Community-based care coordination can improve outcomes and reduce health care costs for children with special health care needs (4). But more research regarding its effectiveness in epilepsy is required (2). The Health Resources and Services Administration funds community-based demonstration projects to improve access to coordinated care for children with epilepsy (2). These projects promote partnerships between health care providers and patients and their families, link care with other community resources, and address barriers to care (2,5).

CDC also supports community-based resources and services for children with epilepsy and their families. Additional information is available at http://www.epilepsy.com/get-help.
